# Effects of Nutrition Education Based on Social Cognitive Theory on Fruit and Vegetable Consumption Among Adolescent Girls in Tanakeke Islands, South Sulawesi

**DOI:** 10.21315/mjms-01-2025-014

**Published:** 2025-08-30

**Authors:** M Bambang Uswatul Firdaus, Healthy Hidayanty, Aminuddin Syam, Veni Hadju, Rahayu Indrisari, Shanti Riskiyani

**Affiliations:** 1 Department of Nutrition Science, Faculty of Public Health, Hasanuddin University, Makassar, South Sulawesi, Indonesia; 2 Department of Health Promotion and Behaviour Science, Faculty of Public Health, Hasanuddin University, Makassar, South Sulawesi, Indonesia

**Keywords:** adolescent girls, fruit and vegetable consumption, nutrition education, undernutrition, social cognitive theory

## Abstract

**Background:**

Indonesia, as an archipelagic country with a vast population, faces persistent challenges of undernutrition, particularly among adolescent girls, which impedes progress toward achieving the Sustainable Development Goals (SDGs). One key issue is inadequate intake of fruits and vegetables (FV). This study aimed to assess the effectiveness of nutrition education (NE) based on the social cognitive theory (SCT) in improving FV consumption among undernourished adolescent girls in the Tanakeke Islands, South Sulawesi, Indonesia.

**Methods:**

A quasi-experimental study through anthropometric and dietary assessments was conducted across four schools. The 12-week NE programme included role-playing exercises, educational modules for students, and monthly parental education sessions. Data were analysed using Statistical Package for the Social Sciences (SPSS) version 28, applying chi-square tests, independent *t*-tests, and paired *t*-tests, and analysis was conducted using repeated measures ANOVA with a significance level set at 0.05.

**Results:**

A total of 88 adolescent girls completed the study. Results showed significant improvements in the intervention group’s knowledge, outcome expectations, self-regulation, and FV consumption (*P* < 0.05). However, no significant change was observed in self-efficacy (*P* = 0.749).

**Conclusion:**

The research shows NE based on SCT, combined with parental involvement, effectively increases FV consumption among undernourished adolescent girls in remote, low-resource areas to improve adolescent nutrition. The lack of improvement in self-efficacy highlights the need for programme refinement to address this construct explicitly.

## Introduction

Indonesia, being an archipelagic nation, has a vast number of natural resources and a large population. However, it requires integrated and extraordinary efforts to manage and build coordinated strategies to achieve the Sustainable Development Goals (SDGs) targets (1). According to the 2018 Global Nutrition Report (2), one in three people suffers from malnutrition, one in 20 children complains of hunger, and one in every five deaths worldwide is caused by poor diet. Developing and low-income countries, especially those in Sub-Saharan Africa, the Pacific region, and Southeast Asia, such as Indonesia, have a higher prevalence of malnutrition (3).

According to United Nations Children’s Fund (UNICEF) (4) data, around a quarter of adolescents aged 13 to 18 years old in Indonesia are stunted, and 9% of adolescents aged 13 to 15 years old have chronic energy deficiency (CED), which means they are thin (body mass index [BMI] < 18.5). In contrast, 16% of adolescents are overweight or obese (BMI > 25). The Basic Health Research report of the Ministry of Health, South Sulawesi Province, revealed that for adolescents aged 13–15 years old, 28.55% were short based on the height-for-age Z-score (HAZ). For thin adolescents based on the BMI-for-age Z-score (BAZ), 10.74% were thin (underweight) (5).

Undernutrition during adolescence, including thinness, stunting (short height for age), and micronutrient deficiencies, has been associated with poor cognitive performance, reduced productivity, and academic achievement (6, 7). A good dietary status during adolescence is essential, as it is the second-fastest growth period after infancy (8). A cross-sectional study conducted by Hidayanty et al. (9) on small islands in Indonesia showed that only 24.5% of adolescents met their calorie intake requirements, and only 13.6% of them met their daily protein requirements. The study also noted that dietary risks, including inadequate fruit and vegetable (FV) intake, were considered one of the primary causes of death for both sexes. According to provincial data, only 4.46% of adolescents in South Sulawesi Province consumed ≥ 5 portions of FV in 2018 (5).

Addressing adolescent nutritional issues requires knowledge and promoting positive attitudes toward balanced nutrition to prevent future malnutrition (10). Transitioning from knowledge to habitual change requires self-efficacy. This concept, developed by Albert Bandura in 1977, is central to social cognitive theory (SCT) and is crucial for behaviour change. Perceived self-efficacy refers to an individual’s belief in their ability to perform a task successfully (11). The development of self-efficacy is influenced by the dynamic interplay of personal, behavioural, and environmental factors, which affect one another and consequently shape health behaviours (12). The research by DiBello et al. (13) provides strong support for including attitudes and self-efficacy in the design and improvement of behaviour change interventions.

Along with knowledge and self-efficacy, Bandura’s SCT includes outcome expectations as key psychosocial factors that influence adolescent dietary behaviour (14). Positive outcome expectations refer to an individual’s subjective perception of the potential benefits of engaging in a specific behaviour (15). The SCT construct also explains that personal factors of self-regulation play a key role in changing an individual’s health behaviour to control their environment successfully, especially when faced with obstacles (16). Environmental influences, such as family support and peer pressure, can either promote or hinder healthy nutritional choices (17). Nutrition education (NE) should target adolescents and their families, as parental habits greatly affect children’s diets (18).

In schools, NE using SCT has been extensively studied and successfully promotes healthy eating habits, such as consuming FV. Several studies have been conducted on this topic (19–23). A systematic review by Luo and Allman-Farinelli (24) revealed that SCT is the most used behavioural theory by dietitians/nutritionists to promote healthy eating interventions. However, the use of this theory is still limited in Indonesia, with only 18.5% of interventions targeting adolescents and 23.1% focusing on FV interventions. Hidayanty et al. (25) recommend using SCT in interventions to improve eating patterns in adolescent girls living on small islands. Therefore, this research aims to implement NE based on SCT on knowledge, self-efficacy, outcome expectations, self-regulation, and FV consumption among undernourished school-age adolescents in the Tanakeke Islands, South Sulawesi.

## Methods

### Study Design and Setting

This study employed a quasi-experimental, non-randomised pre-post test design with a control group. The study was conducted between 1 August and 11 December 2023, in the Tanakeke Islands District, Takalar Regency, South Sulawesi Province, Indonesia (5°30′25.207″S, 119°17′4.469″E), an area characterised by geographic remoteness and nutritional challenges.

### Study Participants and Sampling

In this study, undernutrition was defined using anthropometric standards based on the Indonesian Anthropometric Standard for Children 2020 (26) and dietary intake levels below the recommended age-specific intake according to the Indonesian Recommended Dietary Allowance 2019 (27). Anthropometric indicators included parameters such as mid-upper arm circumference (MUAC), BAZ, and HAZ, while dietary assessments were conducted using 24-hour recall data compared to the national recommended intake levels.

The screening results of the 245 adolescent girls surveyed were used as the basis for determining eligibility and group allocation. This research utilised a hypothesis testing formula to compare the means of two independent populations within a numerical, analytical research framework. The sample size calculation was performed with a significance level (*α*) of 0.05 and a statistical power (1–*β*) of 0.80. For the SCT-based education on FV consumption, the estimated standard deviation (SD) of the mean difference within groups was 0.27, with an expected meaningful difference of X_1_ = 1.47 and X_2_ = 1.25 between the intervention and control groups (28). An additional 15% of participants were included in the calculation to account for potential attrition and maintain adequate statistical power. This is an adjustment made to the educational group sample size to anticipate attrition and ensure sufficient statistical power.

### Sampling and Group Assignment

Cluster sampling was applied by selecting four schools in one district. Two schools were assigned as intervention groups (Public Junior High School 4 Mappakasunggu and Public Senior High School 10 Takalar) and two as control groups (One-Building Junior High Schools Kalukuang and One-Building Junior High School Tompotana). The assignment was non-randomised but based on comparable school size and setting.

### Intervention Description

The intervention group received a 12-week NE based on the SCT programme using the module Becoming Healthy and Active Contemporary Adolescents to Overcome Undernutrition (unpublished data). The SCT group education programme was delivered over six sessions weekly (± 90 minutes per session). Each session began with a previous session review (15 minutes), during which participants reviewed material from earlier sessions and conducted self-evaluations, starting in the second week. Followed by a lecture segment (15 minutes) to introduce new concepts. After the lecture, participants engaged in ice-breaking activities and enjoyed refreshments, which included snacks and fun interactive activities. Next, a 30-minute interactive session was conducted, which involved activities such as discussions, role plays, simulations, or hands-on practice aligned with the session’s theme. The session concluded with a 15-minute Q&A and summary segment, allowing participants to ask questions and revisit key points. In the final session, a dedicated goal-setting segment (15 minutes) was held, where participants were guided in setting personal health behaviour goals to work on before the next meeting. Ice-breaking activities were included two to three times throughout the programme to maintain engagement and prevent boredom.

Adolescent girls were also provided with modules and journals for self-directed learning. Parents of teenage girls also received three educational sessions. Two sessions were conducted face-to-face in a large NE class, and one session for parents via a WhatsApp group. Parents were educated on the impact of environmental factors and observational learning to provide support for increasing knowledge, SCT construction, and FV consumption in adolescent girls through lectures, discussion groups, and experiential learning methods. The positive control group received 12 Ministry of Health leaflets (leaflet KEMENKES) over 12 weeks, accompanied by short 45-minute educational sessions that included lectures, discussions, and ice-breaking activities. Notably, control group parents did not receive any education.

### Data Collection and Tool

The students’ knowledge was assessed using a modified version of the knowledge questionnaire developed by Farisa (29). The instrument consisted of 15 multiple-choice items, each offering five response options (A–E), one correct answer, three incorrect alternatives, and one “I don’t know” option. Correct responses were assigned a score of 1, while wrong and “I don’t know” responses were scored as 0. Scores range from 0 to 15. The study assessed three key constructs from Bandura’s SCT: self-efficacy, outcome expectations, and self-regulation (unpublished data). Self-efficacy is an individual’s belief in their ability to perform a task successfully (11). Outcome expectations refer to the perceived benefits or barriers associated with a behaviour that influences goal-directed actions (30). Self-regulation encompasses planning, setting goals, self-monitoring, making judgements, exercising self-reactive influence, and controlling behaviour (31). Each construct was measured using a validated scale, comprising 11 items for self-efficacy (score range: 11–44), nine items for outcome expectations (score range: 9–36), and 10 items for self-regulation (score range: 10–40). Responses were rated on a 4-point Likert scale, from 1 (“strongly disagree”) to 4 (“strongly agree”).

### Validity and Reliability

The knowledge and SCT construct questionnaires used in this study underwent expert validation to ensure content relevance and clarity. The instruments demonstrated acceptable internal consistency, as indicated by Cronbach’s alpha values of 0.76 for self-efficacy, 0.79 for outcome expectations, and 0.73 for self-regulation. A pilot test was conducted with 30 adolescent girls at Budi Utomo Private High School in Makassar to refine the language and ensure comprehensibility from the target population’s perspective. Feedback from the pilot participants was used to enhance the clarity of the questionnaire items, particularly in terms of word choice and grammar.

### FV Consumption

The FV consumption practices were assessed using a semi-quantitative food frequency questionnaire (SQ-FFQ), which measured average daily intake in grams (g/day). The SQ-FFQ was developed based on definitions in a report by the World Health Organization (WHO) (32), which exclude starchy vegetables such as potatoes, plantains, sweet potatoes, taro, cassava, and breadfruit. The recommended daily intake was set at 400 g/day, comprising 150 g/day of fruit and 250 g/day of vegetables. The questionnaire included 19 types of fruit and 18 types of vegetables, with the option to add additional items if not listed. The SQ-FFQ was validated by experts and adapted with input from teachers and parents to ensure local relevance.

### Statistical Data Analysis

Data analysis was conducted in two phases utilising Statistical Package for the Social Sciences (SPSS) version 28 (IBM Corp., Armonk, NY, US). Categorical variables were reported as frequency counts with percentages (*n* %), while continuous variables were summarised using the mean and SD (mean ± SD). The chi-square test was used to identify significant differences in categorical variables, and an independent samples *t*-test was used to assess mean differences between groups. A repeated measures ANOVA, followed by a Bonferroni post hoc test, was used to compare mean differences within groups from baseline to 12 weeks and between the intervention and control groups. The significance level was set at 5% (two-sided).

## Results

### Participant Characteristics

**Figure 1:**
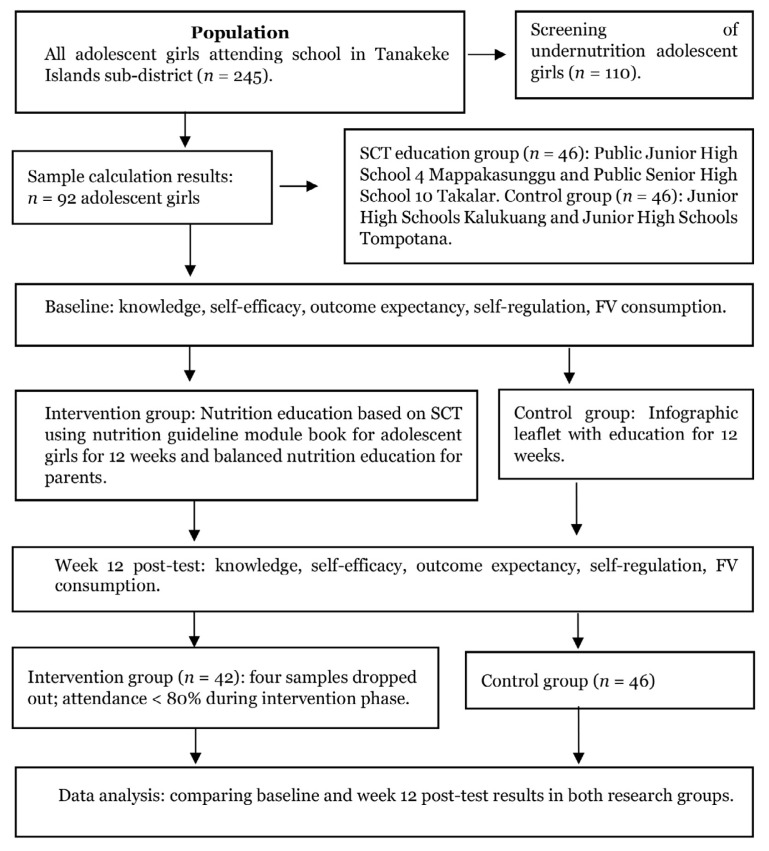
Consort follow diagram of SCT-based intervention on FV

A total of 92 adolescent girls were enrolled in the baseline survey. However, five participants from the intervention group dropped out, leaving 88 girls who completed the 12-week intervention (Figure 1). Most participants were 13 to 15 years old, with a mean age of 13.28 (SD = 1.28). Most parents had only completed elementary school (51.7% of fathers and 52.2% of mothers). The primary occupation of fathers was fishing (74.7%), while the majority of mothers were housewives (86.2%). Notably, most participants came from low socioeconomic backgrounds, with 92.4% of families reporting a monthly income of < IDR 2,000,000. Statistical analysis revealed no significant differences between the intervention and control groups in terms of parental education, maternal occupation, and socioeconomic status. However, significant differences were observed in the age of the adolescent girls and fathers’ occupations (Table 1).

### Intervention Effects on SCT Constructs

**Figure 2:**
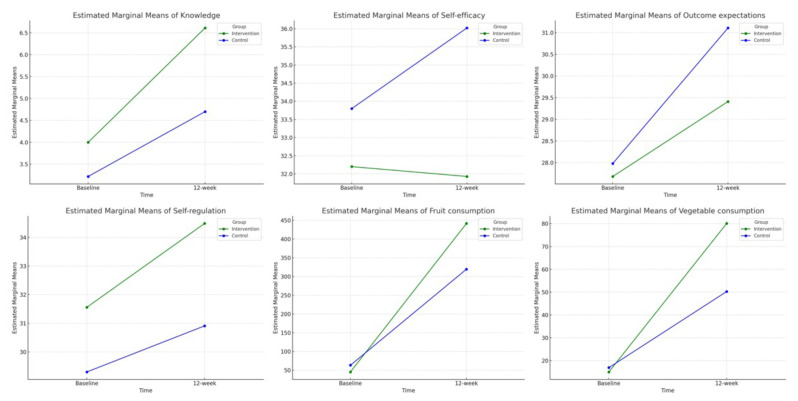
Comparison of constructs of the knowledge and SCT scores and FV consumption change over time between groups

The SCT construct scores between the control and intervention groups at baseline and 12 weeks post-intervention were compared. After adjusting for age and fathers’ occupations, repeated measures ANOVA demonstrated significant between-group differences in self-efficacy, outcome expectations, and self-regulation scores (*P* < 0.001, Table 2). However, no significant between-group differences were found in knowledge scores (*P* = 0.090, Table 2). As illustrated in Figure 2, these changes reflect distinct patterns over time between the groups.

Within-group analyses showed significant improvements from baseline to 12 weeks in knowledge (intervention: mean difference = 2.61; control: mean difference = 1.47; both *P* < 0.001), outcome expectations (intervention: mean difference = 1.73, *P* = 0.018; control: mean difference = 3.13, *P* < 0.001), and self-regulation (intervention: mean difference = 2.92, *P* < 0.001; control: mean difference = 1.60, *P* = 0.048; Table 3). The intervention group showed greater improvements in knowledge and self-regulation. Outcome expectations improved in both groups, with a larger increase in the control group. Notably, self-efficacy did not change significantly in the intervention group (mean difference = –0.26, *P* = 0.748), whereas the control group showed a significant increase (mean difference = 2.21, *P* = 0.039) (Figure 2 and Table 3).

### FV Consumption

After adjusting for age and fathers’ occupations, repeated measures ANOVA showed no significant between-group differences in FV consumption (*P* = 0.063 for fruit, *P* = 0.555 for vegetables; Table 2). As shown in Figure 2, the intervention group consistently demonstrated greater overall improvements. Within-group analyses showed increases in fruit consumption (intervention: mean difference = 395.76 g; control: mean difference = 256.23 g; both *P* < 0.001) and vegetable consumption (intervention: mean difference = 65.04 g; control: mean difference = 33.34 g; both *P* < 0.001) from baseline to 12 weeks (Table 3).

## Discussion

The results highlight the potential of a structured NE programme based on Bandura’s SCT in influencing adolescent girls’ knowledge, outcome expectations, self-regulation, and FV consumption. Notably, while self-efficacy did not improve significantly in the intervention group, it showed significant improvement in the control group. This suggests that the intervention may not have adequately targeted this construct or that external factors in the control setting may have contributed to the improvement.

The intervention and control groups showed increased knowledge regarding FV consumption following the NE programme. This finding aligns with Wulandari et al. (33), who found that SCT education significantly improved knowledge about breakfast among undernourished girls on small islands. Similarly, Mabrukatulhaya et al. (34) reported a significant increase in knowledge about anaemia among girls in the highlands. However, another study based on SCT education noted no significant improvement in knowledge, likely due to its emphasis on physical activity rather than NE (35).

In this study, researchers examined three SCT construction variables, including self-efficacy, outcome expectations, and self-regulation. While outcome expectations and self-regulation improved, self-efficacy did not increase significantly in the intervention group, but it did increase significantly in the control group. This may be due to the intervention’s limited focus on self-efficacy or external influences affecting the control group. The lack of change in self-efficacy might also be explained by high baseline scores and the absence of sufficient mastery experiences and social reinforcement, despite self-efficacy being a central factor in behaviour change within SCT. In the context of health-related processes, self-efficacy is seen as a strong predictor of positive health behaviour change (36). The insignificant self-efficacy in the results of this study is contrary to the majority of NE based on SCT research, such as the results of a survey conducted in Iran (37, 38) and a 12-week intervention study in South Korea (15).

A 12-week NE intervention significantly impacted outcome expectations and self-regulation, but did not significantly affect self-efficacy. Supporting the current study’s results is the research by Zolghadr et al. (39), conducted in Iran to identify the effective factors associated with healthy nutritional behaviour based on the SCT. The findings revealed that while the self-efficacy variable did not show significant results, the variables of result expectations and self-regulation exhibited significant results. The research revealed that self-efficacy did not impact self-regulation, which is consistent with the findings reported by Kim and Han (15). Additionally, Rahayu et al. (40) reported that self-regulation is the most significant determinant of changes in adolescent behaviour when other SCT constructions are unable to play a role in behaviour change.

The study showed that both groups significantly improved their FV intake after participating in the NE programme, with the intervention group achieving even better results. The average fruit intake met the daily requirement (150 g), while vegetable intake remained below the recommended level (250 g). The combined FV intake met the overall guideline (400 g). These results were higher than the average FV intake of 132.4 g/day reported among adolescents in West Java (41). They exceeded the averages found in Jakarta, which were 37.49 g for fruits and 34.2 g for vegetables (42). As a final comparison, a systematic review by Rachmi et al. (43) found that Indonesian adolescents’ FV is generally low (106.6 g/day and 62.1 g/day, respectively) (Table 2).

This research focused on mothers of adolescent girls in the intervention group who received education on balanced diets and nutrition, specifically for adolescents, along with the advantages of consuming FV. The SCT suggests that education acts as an agentic cognitive process realised and comprehended within a social setting like the family, particularly among parents, school peers, and others (44). According to the triadic reciprocal determinism model, the environment can affect the frequency and intensity of behaviours, as the behaviours can also influence the surrounding environment. Influencing a person’s behaviour also depends on environmental factors; for instance, providing FV or new resources that can enhance FV consumption behaviour (45).

The study faced limitations, including participants’ limited basic knowledge and low proficiency in the national language, especially in the control group, where local dialects were common. The timing of the post-test during the fruit harvest also influenced fruit availability. Some adolescent girls also chose not to attend NE during school exams. To address these challenges, the researchers adapted educational materials to local language and literacy levels, used visual and interactive methods, engaged mothers through sessions, introduced online parental meetings, applied cluster sampling, and enrolled extra participants to reduce dropout risk. Careful scheduling helped minimise disruptions during harvest and exam periods.

## Conclusion

In conclusion, this study confirmed that SCT-based NE effectively improves knowledge, outcome expectations, self-regulation, and FV consumption among undernourished adolescent girls in the Tanakeke Islands. The findings underscore the significance of NE-based SCT in enhancing nutrition outcomes among adolescents, particularly in low-resource and remote areas. The study shows that incorporating family-based approaches can enhance intervention effectiveness and highlights the importance of culturally tailored, scalable nutrition programmes. Policymakers should consider using SCT-based frameworks to develop scalable and culturally relevant nutrition programmes.

However, the lack of improvement in self-efficacy suggests that future interventions should include specific strategies such as mastery experiences, peer modelling, and social reinforcement to strengthen this component. Further research is needed to refine these interventions and to specifically focus on increasing vegetable consumption among adolescent girls, especially in island and coastal regions with low- and middle-income populations.

## Figures and Tables

**Table 1:** Demographic characteristics of adolescent girls in the two groups before NE

Variable	Intervention		Control		*P*-value

	Mean (SD)	*n* = 41 (%)	Mean (SD)	*n* = 41 (%)	
Age (years)	13.61 (1.28)		12.98 (1.22)		0.019^a^

Father’s education					
Uneducated		5 (12.2)		12 (26.1)	0.170^b^
Elementary school		25 (61.0)		20 (43.5)	
Junior high school		9 (22.0)		7 (15.2)	
Senior high school		1 (2.4)		5 (10.9)	
College		1 (2.4)		2 (4.3)	

Mother’s education					
Uneducated		11 (26.8)		13 (28.3)	0.882^b^
Elementary school		22 (53.7)		26 (56.5)	
Junior high school		51 (2.2)		4 (8.7)	
Senior high school		2 (4.9)		3 (6.5)	
College		1 (2.4)		0	

Father’s occupation					
Civil servant		2 (4.9)		0	< 0.001^b^
Entrepreneur		1 (2.4)		0	
Fisherman		21 (51.2)		44 (95.7)	
Farmer		14 (34.1)		1 (2.4)	
Unemployed		3 (7.3)		1 (2.4)	

Mother’s occupation					
Civil servant		1 (2.4)		0	0.450^b^
Entrepreneur		1 (2.4)		1 (2.4)	
Fishing		1 (2.4)		1 (2.4)	
Farmer		7 (17.1)		0	
Housewife		31 (75.6)		44 (95.6)	

Socio-economic status					
Low		39 (95.1)		44 (95.7)	0.134^b^
Medium		2 (4.9)		2 (4.3)	

a *t*-test;

b chi-square

**Table 2:** Mean differences of SCT constructs and FV consumption

Variable	Time	Mean^a^ (SD)		Adjusted mean^b^ (95% CI)		*F* stats (df)	*P*-value^c^

		Intervention	Control	Intervention	Control		
Knowledge	Baseline	4.00 (2.16)	3.22 (2.05)	4.00 (3.30, 4.69)	3.21 (2.60, 3.83)	2.94 (1, 84)	0.090
	12-week	6.61 (2.70)	4.70 (2.38)	6.64 (5.74, 7.47)	4.69 (4.00, 5.38)		
Self-efficacy	Baseline	32.20 (4.61)	33.80 (5.74)	32.48 (30.71, 33.67)	33.80 (32.06, 35.54)	37.07 (1, 84)	< 0.001
	12-week	31.93 (4.69)	36.02 (5.70)	32.21 (30.41, 33.43)	36.02 (34.29, 37.74)		
Outcome expectations	Baseline	27.68 (3.32)	27.98 (4.12)	27.76 (26.60, 28.76)	27.98 (8.10, 8.63)	35.78 (1, 84)	< 0.001
	12-week	29.41 (4.73)	31.11 (4.32)	29.40 (28.22, 30.60)	30.91 (29.82, 32.00)		
Self-regulation	Baseline	31.56 (4.73)	29.30 (4.24)	31.45 (30.04, 33.08)	29.30 (28.06, 30.54)	34.64 (1, 84)	< 0.001
	12-week	34.49 (3.89)	30.91 (3.70)	34.62 (33.31, 35.66)	30.91 (29.82, 32.00)		
Fruit consumption	Baseline	45.49 (37.20)	63.38 (59.57)	49.29 (34.18, 57.73)	63.80 (46.35, 80.40)	3.54 (1, 84)	0.063
	12-week	441.72 (150.42)	319.61 (149.83)	442.06 (395.26, 488.17)	319.61 (274.53, 346.69)		
Vegetable consumption	Baseline	15.04 (12.02)	16.92 (21.17)	15.04 (11.17, 18.90)	16.92 (10.61, 23.23)	0.35 (1, 84)	0.555
	12-week	80.09 (65.05)	50.26 (45.94)	80.09 (60.80, 99.37)	50.26 (37.15, 63.38)		

a Descriptive mean;

b based on estimated marginal mean;

c Group-time interaction of repeated measure analysis of variance; CI = confidence interval; adjusted for age (years) = 13.28 and father’s job = 4.18

**Table 3:** Comparison of mean difference of SCT constructs, FV consumption among adolescent girls within each group based on time using repeated measures ANOVA

Comparison	Intervention		Control	

	Mean diff (95% CI)	*P*-value^a^	Mean diff (95% CI)	*P*-value^a^
Knowledge				
At 12-week baseline	2.61 (1.65, 3.56)	< 0.001	1.47 (0.75, 2.19)	< 0.001

Self-efficacy				
At 12-week baseline	−0.26 (−1.94, 1.41)	0.748	2.21 (0.11, 4.32)	0.039

Outcome expectations				
At 12-week baseline	1.73 (0.30, 3.15)	0.018	3.13 (1.93, 4.32)	< 0.001

Self-regulation				
At 12-week baseline	2.92 (1.45, 4.40)	< 0.001	1.60 (0.12, −3.20)	0.048

Fruit consumption				
At 12-week baseline	395.76 (349.11, 442.41)	< 0.001	256.23 (204.70, 407.76)	< 0.001

Vegetable consumption				
At 12-week baseline	65.04 (45.32, 84.77)	< 0.001	33.34 (18.14, 48.54)	< 0.001

a Repeated measures ANOVA; the mean difference is significant at the 0.05 level; adjustment for multiple comparisons: Bonferroni
